# Co-microencapsulation: a promising multi-approach technique for enhancement of functional properties

**DOI:** 10.1080/21655979.2022.2037363

**Published:** 2022-02-16

**Authors:** Iván A. Niño-Vásquez, Diana Muñiz-Márquez, Juan A. Ascacio-Valdés, Juan Carlos Contreras-Esquivel, Cristóbal N. Aguilar, Raúl Rodríguez-Herrera, Adriana C. Flores-Gallegos

**Affiliations:** a Food Research Department, School of Chemistry, Universidad Autónoma de Coahuila, Boulevard Venustiano Carranza E Ing, Saltillo, México; b Tecnológico Nacional de México, Instituto Tecnológico de Ciudad Valles. Ciudad Valles, Slp, México, Ciudad Valles, México

**Keywords:** Microencapsulation, co-microencapsulation, food, polyphenols, probiotics, prebiotics

## Abstract

Co-microencapsulation is a growing technique in the food industry because it is a technique that, under the same fundamentals of microencapsulation, allows the generation of microcapsules with a longer shelf life, using a smaller number of encapsulating materials and a smaller amount of active compounds, while having a greater beneficial activity. This responds to consumer demand for higher quality foods that limit the use of ingredients with low nutritional content and provide beneficial health effects, such as probiotics, prebiotics, vitamins, fatty acids, and compounds with antioxidant activity. The combination of two or more active compounds that achieve a synergy between them and between the encapsulating materials offers an advantage over the well-known microencapsulation. Among the main active compounds used in this process are probiotics, prebiotics, fatty acids, and polyphenols, the main combination being that of probiotics with one of the other active compounds that enhances their benefits. The present review discusses the advantages and disadvantages of the different encapsulating materials and techniques used to obtain co-microencapsulants, where the main result is a higher survival of probiotics, higher stability of the active compounds and a more controlled release, which can lead to the generation of new foods, food supplements, or therapeutic foods for the treatment of common ailments.

## Graphical abstract

**Figure:**
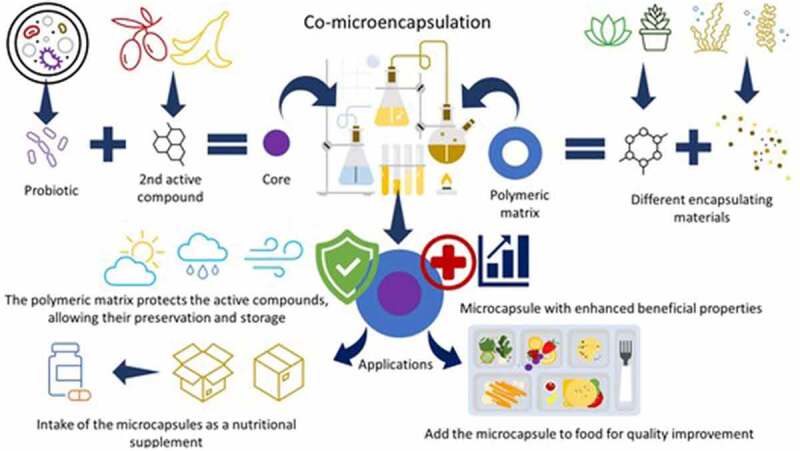

## Introduction

For decades, the food industry has been seeking and developing new technologies to obtain higher quality food in response to consumer demand and the approach of sustainable development goals, such as zero hunger, which consists of providing quality food, health and well-being and, the goal of responsible production and consumption, which considers the reduction of waste generated for example, through the use of agro-industrial waste [1–3].

These higher quality foods are usually achieved by adding ingredients or active compounds that offer health benefits, such as fiber, probiotics, amino acids or essential fatty acids and vitamins; on the other hand, ingredients that could be harmful such as simple sugars, cholesterol, saturated fatty acids and trans fatty acids are reduced [4, 5; 6–11].

The consumption of beneficial active compounds is a way to preserve the health of individuals since the benefit obtained depends on the active compound ingested. For example, fiber consumption helps to reduce serum levels of glucose, cholesterol, and triglycerides, while providing nourishment to the intestinal microbiota and the development of probiotics [4,6,7,11–15]. These bioactive compounds can also prevent or complement the treatment of conditions such as diverticular disease, malabsorption syndrome, diarrheal syndrome, irritable bowel syndrome, intestinal ulcers, hemorrhoids, Crohn’s disease and ulcerative colitis, which are related and/or worsened by low consumption of prebiotics and probiotics or by an alteration of their intestinal microbiota [4,6,7,11,12,14,15]. In the case of diarrheal symptoms caused by an alteration of the intestinal microbiota, present in all ages but predominantly in children under 10 years of age, prebiotics and probiotics can prevent this condition through the promotion of bacteriocin production, by lowering the pH to limit the growth of pathogenic bacteria, and prevent bacterial translocation [4,6,7,11,12,14,15]. On the other hand, in conditions present mainly in adolescents and working population such as irritable bowel syndrome and poor adsorption syndrome there is an alteration of the intestinal microbiota, which needs to be restored to reduce or eliminate the patient’s symptoms, so including prebiotics and probiotics as functional ingredients in food is a coadjuvant treatment option to treat these conditions [4,6,7,11,12,14,15].

The higher quality food industry can also focus on generating substitutes for products with allergenic capacity, such as dairy [4–11,16]. On the other hand, nondairy fermented products have also stood out for their health benefits, such as juices added with probiotics and phenolic compounds. These compounds can be recovered from seeds, shells, bones, scales, among others, decreasing the accumulation of by-products and agro-industrial waste, and their incorporation in food formulations favors consumer health as they help preserve the intestinal microbiota, act as antioxidants and have a lower caloric intake, while their organoleptic characteristics are pleasing to consumers [16].

Despite the known benefits of the different active compounds, their incorporation in food matrices is a technological challenge due to their stability and bioavailability. Therefore, microencapsulation has been used for decades to preserve these active compounds that, exposed to the environment, can degrade in a short time; thus, microencapsulation is a way to prolong their shelf life, either for subsequent consumption dissolved in water or incorporated into a food formulation [15,17–26]. The possibility of combining two or more active compounds in a system to achieve a synergy between them that enhances their beneficial effects while extending their shelf life has led to the strategy known as ‘co-microencapsulation’. In addition to the aforementioned benefits, with co-microencapsulation, production costs are reduced, and it achieves a more significant application of bioactive compounds in the food industry [5,6,24,27–31].

This review article addresses the generalities of microencapsulation and how this technique could become more relevant in the research and production of functional foods with greater stability and bioavailability of the added active ingredients, which serve as oral therapeutic agents for the prevention or treatment of common diseases and everyday pathologies, increasingly frequent due to the current diet and pace of life. Techniques to generate microcapsules, encapsulating materials, active compounds, up to a final focus on the potential benefits and applications of co-microencapsulation are discussed [6,17,23,25,27–29].

## 1. Basic concepts of encapsulation

Encapsulation is defined as the technology in which an active ingredient is contained in a core enveloped by a polymeric matrix that generates a capsule. This active compound must have a beneficial effect and the polymeric matrix is necessary to be administered as a means of transport; this polymeric matrix will be formed by different encapsulating materials [5,17].

**Figure 1.**
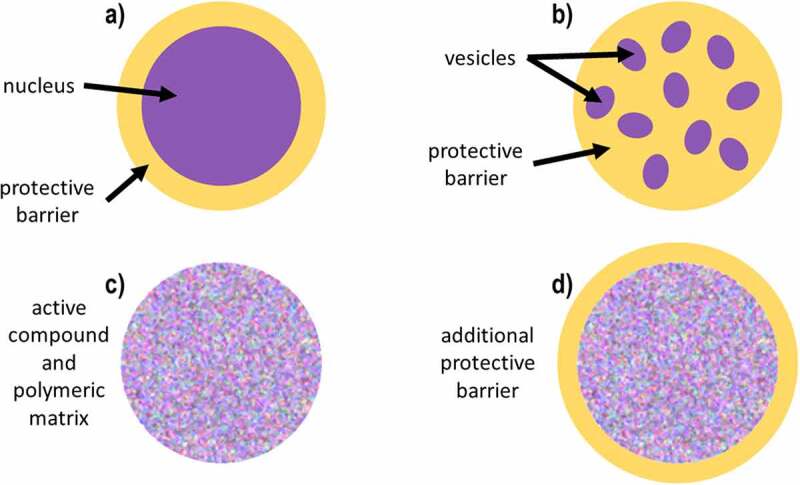
Different types of microcapsules: a] reservoir, a.1) vesicles reservoir, b) inclusive matrix, c) mixed and d) agglomerates.

The capsules generated can acquire different morphologies: reservoir, vesicular reservoir, inclusive and mixed matrix. The reservoir or capsule consists of a single core formed by the active compound, wrapped in a polymeric matrix; this morphology has the polynucleate variant or vesicular reservoir, which consists of multiple cores with the active compound wrapped in a polymeric matrix. The inclusive matrix is a homogeneous mixture of the active compound with the polymeric matrix; such a combination can vary in the ratio of the components, i.e., being 1:1, 1:1.5, 1:2 active compound and matrix, to give some examples. Finally, the mixed morphology consists of a homogeneous mixture of the active compound with the polymeric matrix, i.e. an inclusive matrix. This, in turn, is wrapped by an outer layer of the polymeric matrix. The morphology of the microcapsules depends on the process carried out for the microencapsulation and even the variation in the conditions of the same procedure can give rise to different morphologies. These morphologies are represented in Figure 1 [5,17,32].

Encapsulation has the following objectives 1) to protect the active compound from the environment (light, heat, humidity, acidic medium, etc.) to reduce degradation and prolong shelf life, 2) release the active compound in a controlled manner under certain pH and temperature conditions so that the active compound acts in the desired location, 3) facilitate the handling of the active compound thanks to the protection provided by the polymeric matrix, 4) hide unpleasant tastes or odors of the active compound or any of the encapsulating materials, for which different materials are combined, as well as 5) retain volatile ingredients [17: 5].

Based on the size of the capsules or particles generated, it is referred to as encapsulation, microencapsulation and nanoencapsulation. Each has specific characteristics that differentiate them from each other [33,34].

### 1.1 Microencapsulation

It can be defined as encapsulation in particle sizes between 1 µm and 1,000 µm per unit. These microcapsules have a prolonged release of the active compound, which allows reaching more distal portions of the digestive tract; in this way, once the release process of the active compound is initiated, it can be released gradually, allowing the durability of the effect for a longer time. In addition, it allows the addition of probiotics as active compounds to the microcapsule [23,29,34–36].

### 1.2 Nanoencapsulation

These are particles that are typically between 50 nm and 500 nm in size and have a higher surface specificity, meaning that they have a larger surface area for the same amount of volume. Due to their small size, nanocapsules can be easily distributed homogeneously throughout fluids. At the same time, they have an increased release because the effect of the active compound starts faster than in microcapsules, providing longer durability [33–35,37].

Micro- and nanoencapsulation are usually used to encapsulate a single active compound. However, recently it has been proposed to encapsulate two active compounds. This is called co-microencapsulation or co-nanoencapsulation, with co-microencapsulation being the most studied and used, as it allows the use of a wider variety of bioactive compounds and is more economical [28,29,38].

## 2. Microencapsulation techniques

It is necessary to know the different microencapsulation techniques so that the researchers can decide which method allows them to easily achieve their objectives. The most used microencapsulation techniques are freeze-drying, spray drying, coacervation and extrusion [32,39,40]; each of these techniques will be described in the following section.

### 2.1 Lyophilization

Lyophilization consists of a drying process at a low temperature, usually below 40°C. It is very useful for active compounds and encapsulating materials that tend to be sensitive to heat, limiting their degradation process and achieving a more efficient encapsulation of the active compound and an intact polymeric matrix [41].

In this technique, it is necessary to dissolve the encapsulating materials and active compounds in an aqueous solution until their complete hydration and homogenization. The conditions used are usually 150 rpm at 37°C for 4 hours; then, the solution is sterilized with ultraviolet light and, finally, it is proceeded to lyophilization [38,41].

**Figure 2.**
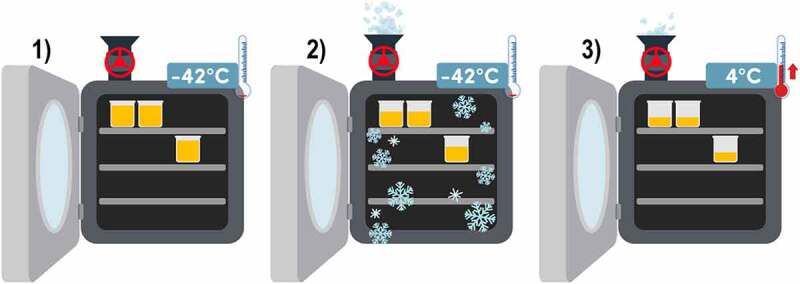
Drying process by lyophilization.

Lyophilization consists of three phases and takes place inside the equipment: 1) which freezes the liquid solution rapidly until solidification, reaching −42°C, 2) then, primary drying removes the frozen water by sublimation, 3) finally, secondary drying removes the rest of the water by raising the temperature from −42°C to 4°C in combination with vacuum conditions, as shown in Figure 2. The freeze-drying process inside the equipment takes about 48 hours; after freeze-drying, the microcapsules should be stored at 4°C for later use. The morphology of the microcapsules is an inclusive matrix consisting of homogeneous distribution of the active compound and encapsulating materials [38,41].

Freeze-drying has certain disadvantages, such as the time involved in the process, as it is slow and requires a heavy investment for specialized equipment that maintains the product under very specific conditions of temperature and pressure; in addition, freeze-drying could result in liquefaction of the product. Another drawback is that freezing of the product is often irregular in some equipment; this can result in particles with different moisture levels in the same batch causing variations in microcapsule quality [41].

However, the advantage offered by lyophilization is that during the process the damage to the active compound and encapsulation material is minimal which also allows working with a wide variety of encapsulation materials; these qualities are shown in Table 1. It differs from coacervation in that the material must be compatible [41].

**Table 1.** Comparison between encapsulation techniques

Technique	Additional materials	Velocity	Advantages	Disadvantages	References
Lyophilization	Specialized equipment	More than 56 hours to get a batch, the batch size depends on the size of the equipment	Minimal damage to ingredients, low water activity	Variations in the microcapsules of the same batch	38, 41
Spray drying	Specialized equipment	12 hours to process 1 liter of solution	Scalable process in processing speed, up to 100 liters/day	Possible degradation of ingredients during the drying process	15, 33, 39, 43
Extrusion	Drying equipment to choose (recommended]	8 hours to process 1 liter of solution [not including drying time)	Minimal damage to ingredients	Without a drying process the shelf life is reduced	45, 46
Coacervation	Choice of drying equipment (recommended], buffers, crosslinking ingredient	4 hours to process 1 liter of solution [not including drying time)	Fast and high industrial scalability	Compatibility between encapsulating materials is required; take into consideration many variables	36, 46–48

Ballesteros in 2017 used freeze-drying as a microencapsulation technique, using as extracts of coffee phenolic compounds as active compounds, and maltodextrin and gum arabic as encapsulating materials; the process lasted 48 hours at −60°C; this process was compared with the spray drying technique under the following conditions: 108 ml/hour with an air inlet temperature of 100°C. The results obtained were that maltodextrin as the only encapsulating material is more efficient for the phenolic compounds of interest and to protect their antioxidant activity, as well as that the freeze-drying technique was more effective compared to spray drying. This improvement occurred because the high temperatures of spray drying degraded some of the phenolic compounds [42].

### 2.2 Spray-drying

Microencapsulation by spray drying consists of high-temperature drying, generally above 100°C, achieving the conversion of a liquid solution into a powder of microcapsules of controlled size and morphology. This is because the entire solution is subjected to the same conditions and procedure, unlike freeze-drying [15,33,43].

Depending on the conditions of the equipment, it may vary in a greater or lesser proportion of the microcapsules produced. Thus, all microcapsules generated by have the same characteristics, even from different batches of the same initial solution [15,33,43].

The procedure consists of preparing an aqueous-based liquid solution in which the encapsulated materials are incorporated with the active compound; this will be introduced into spray drying equipment for microencapsulation, as described by 39: 1) in distilled water, all ingredients should be incorporated at 70°C in agitation at 120 rpm for one hour, 2) then, it is left for 8 hours in constant agitation at 4°C to continue its hydration and incorporation of ingredients, 3) the sample is placed in the spray drying equipment at a rate of 180 mL/ hour, and depending on the amount of aqueous solution available is the amount of time the process will take inside the equipment, 4) finally the microcapsule powder generated by the equipment is collected [15,33,39,43].

**Figure 3.**
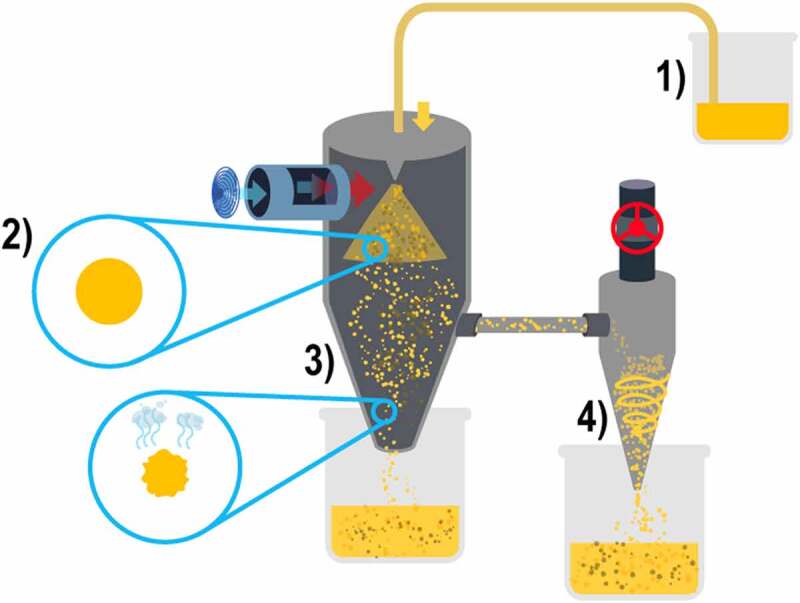
Spray-drying process.

For microencapsulation by spray drying, specialized equipment is needed, which develops the process as follows 1) the previously prepared aqueous solution is placed in the feeding chamber, 2) the solution is atomized in microdroplets in the drying chamber, 3) the drying chamber has a vertical position and with a flow of hot air parallel to the atomizer; this hot air dries in seconds the atomized microdroplets, which fall down the vessel for collection, 4) at the same time, the drying chamber is laterally connected with a cyclone separator that collects most of the generated microcapsules, as shown in Figure 3 [15,33,43].

Laboratory-scale spray drying equipment requires only the setting of the hot air inlet temperature, typically between 100°C and 180°C, the outlet temperature is usually 50°C to 90°C, the air pressure inside the drying chamber ranges from 4 bar to 6 bar, and the atomization rate varies from 100 ml/hour to 1,000 ml/hour. Industrial-scale equipment reaches the atomization rate up to 100 L/hour, making spray-drying microencapsulation a great option for producing health-promoting food products, as it improves production without significant batch-to-batch variability [15,33,43].

The generated microcapsules tend to the morphology of an inclusive matrix because the same solution is used to generate the microcapsules. However, there are some spray-drying equipment with the capability of forming microcapsules with mixed morphology. This is possible because such equipment has two feed chambers; in one of them the solution is placed while in the other the same or different encapsulating materials are placed. Then, in the atomizer the second solution is placed which will surround the first one; this is possible because the atomization of the first solution is done through a pore surrounded by an indented ring where the second solution is atomized at the same time. This is accomplished without changing the conditions of temperature, air pressure, or atomization rate [33,43].

A disadvantage of using spray drying for microencapsulation is that it can damage the active compound and encapsulating materials, mainly due to the high temperatures in the drying chamber, which are shown in Table 2. It has also been described that probiotics tend to suffer mechanical damage due to atomization, in addition to increasing cell permeability, causing the loss of bacterial components and reducing the viability of probiotics [33,44].

**Table 2.** Commonly used encapsulating materials

Material	Structure and source	Properties	Aspects to consider	References
Alginate	Polysaccharide obtained mainly from brown algae	Thickener, emulsifier, stabilizer	Proportion G/M	50
Gelatin	Partial hydrolysis of collagen from pig hides	Gelling agent, high solubility in water	Gelatin type A and B	49, 53
Inulin	Fructan extracted mainly from agave	Prebiotic, slightly sweet taste, provides texture	Polymerization grade	54, 55, 58
Pectin	Polysaccharide obtained from terrestrial vegetables	Prebiotic, stabilizer, gelling agent	Vegetal origin	60–63
Chitosan	Polymer obtained from the chitin of crustaceans	Combination versatility, stiffness, and decreased porosity	Degree of deacetylation, dissolution only in acidic media	64, 67, 68
Whey protein isolate	By-product of milk manufacture	High solubility, gelling,	Bitter flavor	23, 73, 74

G: α-L-guluronic acid. M: β-D-mannuronic acid.

These adverse effects can be reduced by decreasing the hot air inlet temperature, using a double-solution system, increasing the pressure of the drying chamber, and optimizing the composition of the encapsulant matrices, as described by 44, who achieved 100% survival of *L. rhamnosus* CETC 275 microencapsulates using this technology and maltodextrin, sucrose, and whey protein as encapsulant materials, with an inlet temperature of 160°C, and an outlet temperature of 62°C [33,44].

### 2.3 Extrusion

**Figure 4.**
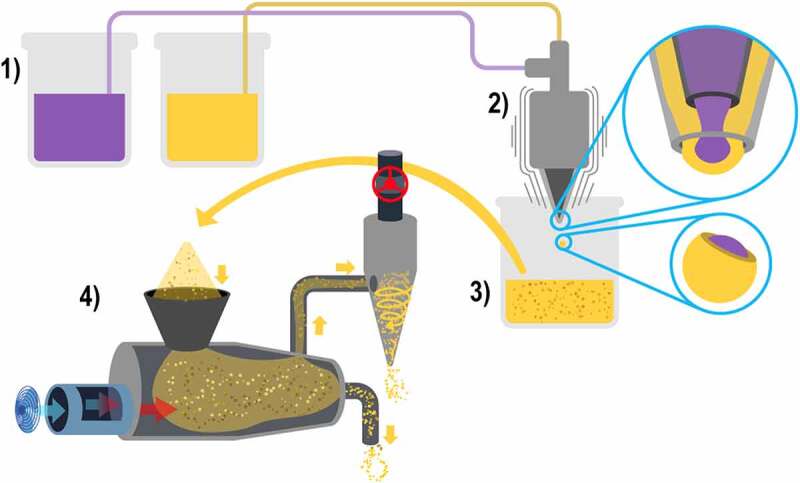
Microcapsule formation by coextrusion.

Microencapsulation by extrusion consists of the following steps 1) all the encapsulating materials and the active compound are mixed in a solution and then placed in a specialized extrusion equipment to form the microcapsules, 2) the extrusion equipment has a pointed nozzle with a specific diameter in which the microcapsules are formed one by one by dripping; however, it is a fast process as the speed of the drip can be adjusted, producing vibrations through the nozzle, creating 100 to 300 microcapsules per second and, 3) these microcapsules fall into a solution that is generally calcium chloride for subsequent collection, resulting in microcapsules with inclusive matrix morphology, as shown in Figure 4 [45,46].

Coextrusion encapsulation is also available, which differs by the use of a concentric nozzle that can process two materials at the same time. The morphology of this type of nozzle is similar to the spray dryer that processes two solutions; this means that it has a central pore with one solution containing the encapsulating materials and the active compound and a ring-shaped indentation surrounding the aforementioned pore. This indentation contains a second solution only prepared with the encapsulating materials, which can be the same ones used in the first solution or can be composed of other materials; the result of coextrusion are microcapsules with mixed morphology [45,46].

Coextrusion provides the microcapsules with an additional barrier that allows greater protection of the active compounds, allowing a longer shelf life and preserving a greater amount of active compounds obtaining a higher beneficial activity for the consumer. Coextrusion compared to extrusion resulted in microcapsules with a shelf life ranging up to 60 days at room temperature and 15–30 days of viability for *Lactobacillus acidophilus* LA [45,46].

The main drawback of extrusion and co-extrusion is that the microcapsules generated usually have high moisture levels, which can compromise their stability, as it increases susceptibility to microbial contamination, resulting in a shorter shelf life that can be less than 30 days. For this reason, fluidized bed drying equipment is recommended instead, which is very similar to the spray drying process, with the only difference being that microcapsules are already formed at lower temperatures; these differences are shown in Table 1 [45].

To extend the shelf life of the microcapsules, they can be dried. It has been reported that adding this step to the extrusion process can reach up to 60 days at room temperature to increase the shelf life of microencapsulates, compared to wet microcapsules that only last 30 days. Generally, the drying process is at room temperature; during the process, the microcapsules flow through the drying chamber in the same direction as the air extracted from the environment. The dried microcapsules fall into a container, while the rest of the solution goes through a cycle to finish drying and can be collected later.

In the study conducted by Silva with the microcapsules dried for 60 days, a higher amount of probiotics (*Lactobacillus acidophilus* LA) was also observed. These benefits are also reported in coextrusion with or without drying [45].

### 2.4 Coacervation

Coacervation is defined as the process of association of solutions and/or macromolecules with opposite charge between them, e.g. proteins, polymers, polysaccharides. This association seeks an improvement with the change of the environment, such as pH, temperature and available ions in the solution. Depending on the number of encapsulating materials, it is defined as simple coacervation or complex coacervation [36,46–48].

Simple coacervation involves a single encapsulant material that achieves its coacervation by dehydration of the solution, whereas complex coacervation involves more than one encapsulant material, which achieves coacervation by the electrostatic interaction of their charges [36,46–48].

Coacervation is useful for micro- and nanoencapsulation, giving two types of morphologies, mono- and polynucleated as the encapsulating materials are placed surrounding the active compound. The main compounds used for complex coacervation are polysaccharides and proteins; this is because both are polymers with opposite charges that form a matrix surrounding the nucleus [36,47,48].

Some of the most commonly used proteins are gelatin, whey protein, egg albumin or even vegetable proteins from soy, pea, chia and wheat, while the most commonly used polysaccharides are usually alginate, chitosan, pectin, carboxymethyl cellulose and gum arabic [36,47,48].

**Figure 5.**
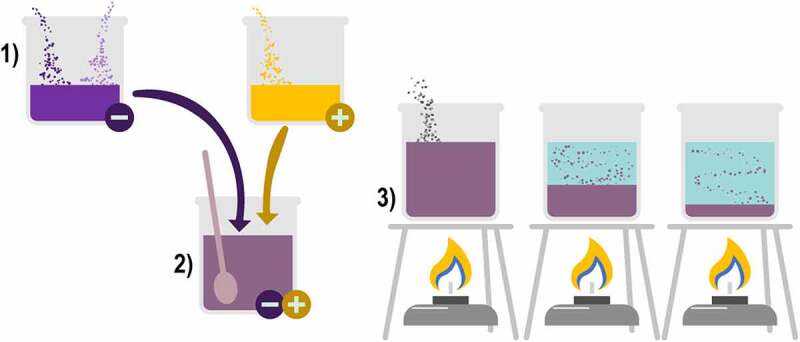
Microencapsulation techniques by coacervation.

For complex coacervation to occur, three main ingredients are needed: 1) the solvent, usually an aqueous solution, 2) the active compound and 3) the encapsulating materials. The preparation requires four steps: 1) an anionic solution is prepared with the encapsulating materials and the active compound to be encapsulated, 2) within a different vessel, a cationic solution is prepared with the protein encapsulating material and both solutions are mixed until homogenized, 3) changes in pH, temperature or salinity are applied so that coacervation occurs and the two solution phases are separated: the coacervation phase at the bottom of the vessel is the one with a higher amount of the encapsulating materials and the active compound, and the equilibrium phase has a lower concentration of those, the difference between phases occurs because the encapsulating materials coacervate around the active compound, thus producing the settling of the formed nucleus, 4) finally in order to build the particles, the temperature is changed or a crosslinking ingredient is added, this stimulates the production of bonding between the encapsulating materials, as shown in Figure 5 [36,47,48].

Three main methods are commonly used for drying microcapsules from aqueous solution: freeze-drying, spray drying, or cold drying. The latter consists of equipment that, through temperature changes, evaporates the solution and removes water by condensation [36,47,48].

Among the parameters that can vary during the coacervation process are pH, temperature, ionic strength of each encapsulating material, compatibility of the encapsulating material, concentration of each encapsulating material, number of salts added and homogenization speed of the encapsulating materials and the active compound. All these variables tend to condition the efficiency of the process, so it is necessary to investigate the optimal conditions for each encapsulating material as well as to search for the best materials to use for coacervation [36,47,48].

However, once the methodology for encapsulation is selected, complex coacervation is highly reproducible and affordable, as shown in Table 1. This is because highly specialized equipment is not needed, and efficient encapsulation of the active compound (>99%) can be achieved, limiting compound wastage, resulting in microcapsules of nucleated morphology. Moreover, the generated microcapsules are projected to effectively have high protection against the environment [36,47,48].

Almeida Paula et al. in 2019 used coacervation as a technique for microencapsulation of *Lactobacillus plantarum* as active compound and gelatin and gum arabic as encapsulating materials; as a drying process, they described a freeze-drying technique. Using this methodology, 97.8% efficiency in microencapsulation was reported, as well as 80.4% viability of the microorganisms after simulated *in vitro* digestion, while free bacteria only reached 25% viability after the same treatment. It was also reported that after storage for 45 days at 8°C, there was no significant decrease in bacterial viability [49]. From this study also derives the recommendation to store microcapsules at temperatures below 8°C to prolong their shelf life. With storage at low temperatures, it is possible to extend the shelf life up to more than twice as long compared to microcapsules stored at room temperature (25°C), as well as maintain the benefits of the active compounds without significant reduction from day zero to day 45. Storage at 8°C and −18°C did not show significant changes in the activity of the active compounds, which represents an advantage by not requiring equipment to maintain the frozen microcapsules [49].

## 3. Encapsulant materials

Encapsulating materials are defined as those ingredients that can be used to give some structure to microcapsules and within the ideal properties they should have are that they should be biodegradable, inert (should not interact with the active compound during processing or storage), should ensure protection against the environment (humidity, light, heat, oxygen, etc.), be malleable during different procedures or conditions and be approved for use in food. Additionally, it can come from elements available in food, limiting the use of synthetic materials in its processing, and have adhesion characteristics to the gastric mucosa to increase the bioavailability of the active compound. This last feature is relevant as it could reduce the amount of active compound administered but maintain its activity, efficiency, and efficacy.

Finally, the economic aspect is the main limitation to achieve the ideal properties of encapsulant materials. Therefore, it is a challenge not to increase production costs by improving encapsulant materials; however, this increase could be justified by demonstrating the benefits provided by encapsulant materials [33,35,42]. One of the cheapest ways to improve ideal properties is by producing smaller capsules (microcapsules and nanocapsules), which have a larger surface area that improves bioadhesion, thereby increasing the bioavailability of the active compound. This also allows using less amount of the active compound [5,17,32,35,42].

### 3.1 Alginate

Alginate is a polysaccharide formed by α-L glucuronic acid (G) and β-D mannuronic acid (M) linked by 1–4 glycosidic bonds; these polymers are grouped in a straight line without any branching. It is obtained from brown algae, mainly *Laminaria hyperborea, Macrocystis pyrifera* and *Ascophyllum nodosum*; each species produces different polymers, and the proportion of each polymer determines the physical and chemical properties. It can be found in salt form: sodium alginate, potassium alginate, ammonium alginate and calcium alginate, with sodium alginate being the most commonly used [32,50,51]. Alginate is biodegradable, and humans cannot metabolize it; it is considered GRAS (Generally Recognized as Safe) by the FDA and the European Commission authorizes it as a food additive [40,50,].

Sodium alginate is used in the food industry as an encapsulating material as it provides thickness, stability and gelling to microcapsules. These characteristics will depend on the number of polymers: if the proportion and length of the glucuronic acid block are higher, denser, and stiffer gels are generated, as shown in Table 2. This makes them more effective against moisture, while a higher amount of mannuronic acid produces more flexible and porous gels [32,50].

Alginate, mixed with other encapsulating materials, is the most recommended and used in the food industry (mainly for micro- and co-microencapsulation). However, despite being an excellent material to protect the active compound from the environment, it limits in vitro and in vivo release and does not favor the preservation of probiotics. Therefore, it is often combined with two or more encapsulating materials, such as inulin, whey protein, chitosan, gelatin, and pectin. This provides a prebiotic source for probiotics and adhesion to the gastric tract and facilitates a faster and more efficient release of the active compound [14,40,50].

Nami et al. in 2020 optimized a polymeric matrix of alginate, Persian gum, and inulin for microencapsulation of *Lactococcus lactis* ABRIINW-N19, and then added the microcapsules to commercial orange juice without preservatives. The optimal polymeric matrix reported consisted of 2% inulin, 1.5% alginate, and 0.5% Persian gum. This matrix protected against in vitro digestion, allowing a survival of over 84% compared to 33% for free bacteria. After adding the microcapsules to the orange juice, it was stored at 4°C, and evaluations were performed every 7 days until day 42. It was found that there were no significant changes in pH, an increase in the number of bacteria through the passage of days in storage, reducing Brix and malic acid [52].

### 3.2 Gelatin

It is a proteinaceous material obtained by partial hydrolysis of collagen. Depending on the type of hydrolysis performed, the type of gelatin will be generated. Type A gelatin is obtained by acid hydrolysis, mainly from pigskin, and type B gelatin is obtained thanks to alkaline hydrolysis, mainly from animal bones and skin [49,53].

Recently, the use of alternative sources of collagen has been proposed, such as aquatic animals, taking advantage of most animal parts such as skin, bones, scales and fins of fish; this due to cultural, ideological and religious issues. Acid hydrolysis is usually used in these aquatic animal products. Gelatin obtained from marine animals is of higher cost than that obtained from mammals and is recovered in smaller quantities [49,53].

Gelatin is commonly used with alginate for microencapsulation. It can be easily associated with the formation of gels with high gastric strength, resulting in increased survival ability of probiotics, with unencapsulated survival values of 50.36% after *in vitro* digestion increasing to 85.39% with the use of an alginate (1.35% w/v) and gelatin (0.65% w/v) matrix. Vaiziri in 2018 described the microencapsulation of *Lactobacillus plantarum* in a biopolymeric matrix of 13 variants in the amount of alginate, pectin, and gelatin, using extrusion as a microencapsulation technique without drying process. The results were that microcapsules made of alginate as the sole encapsulating material had the lowest degree of viability (79%). However, they provided effective protection against *in vitro* digestion. Therefore, the encapsulating matrix described as optimal is composed of 1.06% alginate, 0.55% pectin and 0.39% gelatin with an encapsulation efficiency reaching 97%, in addition to achieving effective protection against *in vitro* digestion [28].

### 3.3 Inulin

Inulin is a polymer that belongs to the non-digestible carbohydrates, specifically, fructans. It is formed by fructose units joined together by β-bonds; the variations that each type of inulin presents is its degree of polymerization. Inulin can be found naturally in agave, yam, garlic, artichoke, onion, asparagus, among others [54,55].

Inulin has a prebiotic effect on *Bifidobacterium, Bilophila* and *Anaerostipes*, specific to the human microbiome. It is therefore considered a source of fiber, which also has an antioxidant activity slightly lower than that of vitamin C. Moreover, it dissolves moderately in water without exhibiting precipitation, and is not affected by pH in a medium ranging from 4 to 9. These characteristics make inulin an ideal ingredient to be used as an active compound and/or encapsulating material; although most articles on microencapsulation only consider it either as an active compound or as an encapsulating material [54–58].

As an encapsulating material, it is used to give texture and a slightly sweet taste, approximately 10% of the sweetness given by sucrose. However, it can replace up to 35% of sucrose molecules without significantly affecting the flavor. It is also an excellent substitute for fats and sugars as it only provides 25% to 35% of the caloric contribution of glucose; these qualities are shown in Table 1 [54,55,57,58].

Verruck et al. in 2017, encapsulated *Bifidobacterium animalis* subsp *lactis* BB-12 within a matrix of inulin and goat whey protein, using the spray drying technique achieving 95% survival of the bacteria. Nunes et al. in 2018 used spray drying as a microencapsulation technique, achieving a survival reaching 89% for *Lactobacillus acidophilus* LA-5 in a polymeric matrix of gum arabic, maltodextrin and inulin[33,59].

### 3.4 Pectin

This is a heterogeneous family of polysaccharides in plants. This polysaccharide is structurally complex and is formed by multiple polysaccharides such as galacturonic acid (GaIA), homogalacturonan (HG), rhamnogalacturonan I (RG-I), and rhamnogalacturonan II (RG-II). Likewise, some authors suggest the presence of other polysaccharides such as xylogalacturonan and apigalacturonan, without also addressing rhamnose, apiose, fucose, acetic acid, galactose, arabinose, xylose, glucuronic acid, deoxyloxo-hepta-tosaric acid, and deoxy-manno- octulopyranosilonic acid [59–63].

The structure of pectin varies depending on the source. Despite this, the physicochemical properties are similar among the different pectins, as they all consist of 65% linear GalA homopolymer bound to HG, while RG-I and RG-II account for about 25% and 15%, respectively. Such polysaccharides are covalently bound since when seeking to isolate each component, aggressive chemical treatment using multiple digestive enzymes is required [59,60,62,63].

The benefits that pectin has demonstrated are the reduction of serum cholesterol and glucose levels, prebiotic activity in the colonic microbiome, with pectin being metabolized by probiotics to produce short-chain fatty acids. In addition, pectin has also been shown to have anticarcinogenic activity. However, having structural changes from one pectin to another, depending on the source, makes it more challenging to determine which structure would be ideal to exert biological effects as an anticarcinogen [59,60,62].

In microencapsulation, pectin is used to provide stability to microcapsules. It has the gelling ability, which allows the use of probiotics as an active compound; the gelling capacity is directly proportional to the amount of esterified units of GalA, this advantage is compared in Table 2. Typically, pectin is classified depending on the esterification of GalA; high degree of esterification >50%, and a lower degree <50% [60–62,64–66].

Pectin compared to alginate shows higher resistance to i*n vitro* digestion, protecting the encapsulated active compound more efficiently; however, alginate protects the active compound better against the environment, allowing storage for longer periods than those encapsulations made with pectin. This is because pectin generates a very porous microcapsule. Therefore, as with other encapsulant materials, it is recommended to combine more encapsulant materials; pectin is often combined with alginate, chitosan, inulin, and gelatin [61,62].

Zhang in 2015 reported 90% survival of *Lactobacillus salivarius* NRRL B-30514 in a matrix of whey protein, anhydrous milk fat and pectin using the spray drying technique. The same authors, using the same methodology and the same bacteria, reported in 2016 78% to 86% survival using a polymeric matrix of soybean and sugar beet pectin [33].

### 3.5 Chitosan

Chitosan is a polymer obtained from chitin, which can be found in the shells of crabs and shrimp, and in krill shells, although chitin can also be found in exoskeletons of fungi, arthropods and insects, cephalopod skins, and mollusks. Chitin is the second most abundant natural polymer, behind only cellulose [64,67–69].

Once chitin is obtained, it undergoes demineralization, deproteinization, and deacetylation processes; all these processes are usually carried out using acidic solutions and basic solutions that result in the creation of chemical waste, an alternative is the use of ultrasound for the deproteinization process, thus limiting the generation of waste, while being a fast technique with a high potential to be scaled up to industrial level [69]. Depending on the efficiency and quality of these processes, it will provide chitosan with different characteristics, the main one being the degree of deacetylation which is directly proportional to the cost and quality of the chitosan produced [67–69].

Chitosan is composed of glucosamine and N-acetylglucosamine with a high abundance of reactive hydroxyl and amino groups, which allows the formation of hydrogen bonds resulting in the formation of crystals and reactive groups, increasing the polarity and the degree of electrostatic repulsion, resulting in a higher solubility than chitin. However, despite this, chitosan is only soluble at pH below 7 [67,68].

Typically, commercially available chitosan varies from 40% to 98% in the degree of deacetylation; chitosan with a degree of deacetylation exceeding 83% is frequently used. The higher the degree of deacetylation, the greater the flexibility of the molecules, viscosity, solubility, but the lower the adhesion of the gastrointestinal mucosa; these qualities are compared with the other materials in Table 2. It is also a nontoxic material (corroborating the intake of 6 grams per day), odorless, biodegradable and biocompatible. This is because it degrades slowly into amino sugars within the gastric pathway [67,68].

Due to the reactive capacity of chitosan, it has been proven to bind to negatively charged lipids, preventing their absorption to be subsequently discarded, reducing serum lipid levels, mainly cholesterol. In addition, this reactivity can make it behave as an antacid, since it dissolves in gastric acid (demulcent capacity), subsequently binding to those acid molecules [67].

As an encapsulating material, it can be used to provide an impermeable barrier because it needs an acidic solution to dissolve it, limiting the exposure of the active compounds to the environment. Krasaekoopt and Watcharapoka, in 2014, used alginate for co-microencapsulation of Lactobacillus acidophilus and Lactobacillus casei as a probiotic source, and galactooligosaccharides (GOS) and inulin as a prebiotic source. Subsequently, an outer layer of chitosan was added at concentrations of 0.5%, 1.0% and 1.5%, being a mixed encapsulation type [67,68].

*Lactobacillus acidophilus* encapsulated together with GOS and *Lactobacillus casei* encapsulated with inulin showed that a higher amount of chitosan better protects the active compounds when subjected to in vitro digestion. Compared to other combinations that had inconclusive results, the authors refer that such effectiveness is due to the added prebiotic; no conclusion is made about the different proportions of chitosan used [67,68].

The hydroxyl and amino reactive groups of chitosan allow it to be added with other molecules to form new compounds, such as hydroalcoholic extracts (HAE) of different plants like ginger, rosemary and green tea. HAEs combined with chitosan show more excellent protection against oxidative processes of the active compounds. The new matrix showed higher water solubility; it also improved the biodegradation of chitosan. This matrix could be incorporated as an encapsulating material in microcapsules generated with low moisture content [68].

There are several reports on different mixtures of chitosan, with cellulose derivatives (carboxymethyl cellulose, methyl cellulose), cinnamaldehyde (cinnamon extract), sodium sulfate and turmeric (herbaceous plant), propolis (resin produced by bees), olive pomace, among others. Each of these new matrices demonstrated an improvement in characteristics such as increased rigidity, barrier property against light and oxygen, limitation of oxidation of the active compounds, as well as changes in water solubility. The great versatility of chitosan to be combined opens an area of great opportunity for the research of new matrices for encapsulation with all kinds of products of nutritional nature [68].

Using the coextrusion technique, the optimization of a polymeric matrix for the microencapsulation of *Bifidobacterium animalis* subsp. *lactis* BB-12 was achieved, using sodium alginate in a constant amount for the subsequent addition of chitosan in different amounts, limiting the porosity of the generated microcapsules and protecting the active compound more efficiently. 1.5% alginate was used as a control, and the tests were performed with 1.5% sodium alginate plus chitosan [46]. Chitosan was added at a ratio of 0.1% in each trial until 0.5% was reached; the results obtained were that the encapsulation efficiency is improved by adding 0.4% chitosan compared to the other microcapsules generated. The alginate control had a higher encapsulation efficiency reaching 87% while the alginate plus 0 4% chitosan assay reached 89%. This can be attributed to the decreased porosity of the microcapsules; when 0.5% chitosan or more is added, the encapsulation efficiency is reduced due to the increased stiffness in the encapsulant matrix, preventing the release of bacteria for enumeration [46]. Then, the microcapsules were evaluated in *in vitro* digestion, reporting a survival of 83%, a better result than the 71.9% obtained for free bacteria [46].

### 3.6 Whey protein isolate (WPI)

Whey protein isolate is obtained as a by-product during cheese making; for every ten liters, nine will be whey. It is formed after the pasteurization process; in this step lactic acid bacteria and rennet (a set of different enzymes) are added to coagulate the mixture and form cheese. Whey can also be extracted as a by-product of casein manufacture and certain fermented milk beverages [70–73].

Whey obtained by the conventional method has a high number of proteins such as albumin, β-lactoalbumin, immunoglobulins and β-lactoglobulin, as well as the essential amino acids: leucine, isoleucine and valine, proteins constitute more than 90% of whey. Whey is an ingredient in milk drinks or can be added to food for athletes or people trying to lose weight, as its proportion of lipids is negligible (0.4%) [70,73,74]. It is also an alternative for lactose-intolerant people (<0.5%) as it has a limited amount of lactose. Normally, beverages and foods made with whey usually have a slightly bitter taste; this is attributed to the pH (below 6.0) because the isoelectric point of whey is 5.1, and its usual pH is 4.6 [70,73,74].

It is used as an encapsulating material in co-microencapsulation as a protein source for probiotics, being easily incorporated due to its high solubility in water, resulting in high encapsulation efficiency to subsequently gel the generated microcapsules, as shown in Table 2 [23,73,74]. In general, with microencapsulation, bioactive compounds are intended to be released in the small or large intestine to exert their beneficial effect. However, in an *in vivo* digestion model, microcapsules generated with whey would release the bioactive compound in the stomach due to the high solubility of whey in acidic pH [23,70,74].

The gelation capacity of whey can be modified with a pre-gelation treatment prior to microencapsulation. This can be achieved with Maillard reactions, which consist of the addition reaction of an amino group of a protein and a carbonyl group of sugar; the Maillard reaction is also known as glycosylation reaction or browning reaction, the latter name is due to the dark color the ingredients take on [23,73,74]. Heating whey at 80°C for 2 to 5 h improves the gelling ability; the duration of the treatment depends on how much one wants to increase the gelling ability. Modification of whey protein isolate decreases its solubility (due to protein denaturation), improves encapsulation efficiency, and provides enhanced digestibility resistance, continuous and controlled release of active compounds, and improved storage stability [23,70,74].

The application of this Maillard reaction pregelation method has been used to microencapsulate the probiotic *Bifidobacterium animalis* subsp *lactis* INL-1 in a whey matrix with dextrans of different molecular weight (6 kDa, 70 kDa, and 450 kDa), in a weight ratio of 1:0.6 (whey predominating). To carry out the Maillard reaction, each matrix combination was heated at 60°C for 5 days at 65% relative humidity to subsequently encapsulate the probiotic [23]. The reported results are that microencapsulation after Maillard reaction of the combination between whey and dextrans offers greater protection to *in vitro* digestion than the control having only whey without pregelation treatment. Among the different combinations of dextrans; dextrans with a molecular weight of 6 kDa showed greater protection against *in vitro* digestion and maintained higher viability of probiotics when stored at 25°C from the sixth month and up to 12 months. In contrast, storage at 4°C did not differ significantly between the different matrices [23]. Khem et al. in 2016, using the spray-drying technique, reported a 25% to 69% increase in survival of *Lactobacillus plantarum* A17 using WPI as the sole encapsulating material, while Ying et al. also in 2016, using the same technique, reported 80% survival of *Lactobacillus rhamnosus* in an encapsulation matrix of whey protein, glucose, and starch [33].

## 4. Co-microencapsulation

The main application of co-microencapsulation is the combination of probiotics with another active compound, mainly prebiotics, lipids, and more recently, polyphenols, to mention a few. This is done with the objective that the probiotics interact with the other active compound to enhance their beneficial effects or also for the probiotics and the added active compound to exert the same benefit through a different mechanism [6,24,28,38].

### 4.1 Probiotics

Probiotics are nonpathogenic microorganisms that must survive in some food product for ingestion, survive through the gastric tract, bind to the gastrointestinal mucosa, colonize the gastric epithelium, and benefit the host [75–78]. The benefits of probiotics are strain-specific, so the effects are not extrapolable to other microorganisms, even if they are of the same species [79].

The combination of probiotics with these active compounds has two main objectives, in addition to those already mentioned. One is to increase the survival or viability of the probiotics themselves after storage and/or digestion, and the second is to enhance the benefits of both active compounds. This can be achieved by increasing the viability of the probiotics, as well as by the prebiotic effect that the added bioactive compounds can have, allowing to accelerate their reactivation [12,24,28,38,76].

It is worth mentioning that the most used probiotics belong to the *Lactobacillus* and *Bifidobacterium* species. The following is a description of the main active compounds with which probiotics are combined to have a greater benefit, either greater encapsulation efficiency, greater survival to in vitro digestion, longer shelf life, greater probiotic activity, among other benefits of each combination of active compounds [7,23,76,80,81].

### 4.2 Prebiotics

Prebiotics are substances that exclusively metabolize probiotics to carry out their functions, just as once some prebiotics have been metabolized, they can be assimilated by humans. Prebiotics have very specific benefits, so the functions that a prebiotic has with a particular microorganism are not extrapolable to all microorganisms [24,58,75,82,83].

In 2020, the use of mannitol as a prebiotic for co-microencapsulation together with *Bifidobacterium animalis* subsp. *lactis* BB-12 (probiotic) was evaluated. The results indicated that the use of mannitol improved the efficiency of microencapsulation, having 75.54% in the control (without mannitol) and up to 94.15% efficiency in the one using mannitol; however, as the amount of mannitol increased, survival decreased. This is because the increased mass in the microcapsules reduces the space available for bacteria to be added to the microcapsules [46]. In addition, the survival of bacteria after *in vitro* digestion was better in those microcapsules to which mannitol was added. This in vitro digestion was also carried out with free, non-encapsulated bacteria, where bacteria to which mannitol was added also had a higher survival than those that were not encapsulated [46].

In a polymeric matrix of alginate and Persian gum, Nami et al. evaluated the addition of inulin to improve the survival of *Lactococcus lactis* ABRIINW-N19 in *in vitro* digestion. They generated different microcapsules by adding inulin at 0.5% for each test without reducing the previous amount of alginate and Persian gum, 1.5% and 0.5%, respectively [52]. As mentioned in section 3.1 (alginate), these microcapsules were added to a commercial juice without preservatives. When the juice was analyzed, a more significant number of probiotics were obtained than the inoculated ones, as well as a lower amount of Brix degrees without altering the pH [52].

### 4.3 Lipids

They are a very varied group of compounds, among which fats, oils, steroids, and waxes stand out; the compounds that are part of the food industry are fats and oils. Lipids have common properties, such as being insoluble in water, a high energetic contribution, dissolve certain vitamins, as well as there are some that we must ingest due to the inability to synthesize them, such as essential fatty acids [84]. Lipids are important in the diet and in the physiology of the human body since they are the main ones involved in diseases such as overweight, obesity and diabetes mellitus; as well as in atherosclerosis, hypertension, acute myocardial infarction, and cerebral vascular events (CVD) [84].

The fatty acid that has been studied the most is docosahexaenoic acid, better known as DHA, which is synthesized from α-linoleic acid and obtained directly from fish oil. DHA is necessary for brain and retinal development [84]. In 2018, an article was published using an oil that had a combination of fatty acids: DHA (58%), oleic acid (16%), palmitic acid (15%), myristic acid (5%), and eicosapentaenoic acid (1%). This oil was used in combination with *L. plantarum* (probiotic) to generate microcapsules [28]. Vaziri concluded that microcapsules with DHA managed to protect probiotics more efficiently subjected to in vitro digestion; moreover, DHA gave them more thermal stability (according to thermogravimetric analysis, TGA), compared to microcapsules without DHA. However, the study refers that viability after storage was not evaluated, an important parameter to evaluate since fatty acids are susceptible to oxidation, therefore, they would alter the viability of the probiotic used [28].

In 2017, Errate et al. generated microcapsules of *Lactobacillus casei* and omega-3-rich tuna oil wrapped in a polymeric matrix of whey, milk protein and gum arabic, using the complex coacervation technique for subsequent spray-drying and storage at 4°C. The results reported that microcapsules with both active compounds had a two-fold higher survival in *in vitro* digestion compared to microcapsules with only the probiotic without the omega-3 fatty acids, while free bacteria subjected to in vitro digestion lost their viability [27].

### 4.4 Polyphenols

Polyphenols are molecules that originate as part of plant metabolism. They are characterized by having several phenolic rings in their structure and at least one hydroxyl group. Polyphenols provide plants with part of their aroma and color, in addition to other properties conferred to plants, such as antioxidant, antimicrobial and anticancer activity [12,17,42,85]. It has also been observed that they can be used as a source of prebiotics since in limited amounts, they do not show inhibition of probiotic growth; on the contrary, they stimulate the growth of probiotics [12,17,42,85].

Polyphenols are classified into several groups; each group varies from one plant to another, as well as the amount and the place in the plant where it is located. They can be in the roots, in the stem, in the leaves or in the fruit, but they are normally located in the fruit, and this is where most studies and extractions are carried out since it does not damage the plant and allows continuous production. In the fruit they can be in the peel, pulp and/or mesocarp [17,85,86]. Because polyphenols are susceptible to degradation by exposure to the environment, they are susceptible to degradation by indirect light. For this reason, encapsulation is a widely used method to preserve them for subsequent use in the food industry [17].

Authors [87,88] evaluated the functional properties of co-microencapsulation of anthocyanins (polyphenol) extracted from carnelian cherry and *Lactobacillus casei* (probiotic), to evaluate their properties after storage for 90 days, as well as their application in a yogurt. The results after 90 days of storage were as follows: antioxidant activity showed no changes, remaining at 54.43 mMol Trolox/g, total anthocyanin content did not decrease, remaining at 19.86 mg C3G/g, probiotic content decreased from 9.39 × 10 × 10^9^ CFU/g to 4.41 × 10^9^ CFU/g [38]. These microcapsules were added at 2% and 5% of the total container to two yogurt samples that were stored at 4°C for 21 days. The authors report that the 2% sample showed a decrease in anthocyanin content and antioxidant activity, while the sample added at 5% showed no significant changes. In addition, *in vitro* digestion degraded anthocyanins up to 37% after 2 hours [38].

When probiotics are used in microencapsulation, it is also recommended to store the active compounds at temperatures below 8°C, to limit the metabolic activity of probiotics, and better preserve the other active compounds and encapsulation materials [49].

## 5. Co-microencapsulation in the bioengineering field

In addition to many of the benefits mentioned above, the field of bioengineering could greatly benefit from the implementation of co-microencapsulation methodologies. Micro- and co-microencapsulation have been shown to improve the thermal stability of certain substances such as aromatic oils that can be used as an antibacterial agent to improve the shelf life of some foods without affecting the odor, taste, or any other of the food properties [89–91]. An example of these oils is thymol. In 2019 Zhu, Z et al. demonstrated that the use of a microencapsulation methodology for thymol oil prevented the growth of *Escherichia coli* and *Staphylococcus aureus* in naturally contaminated milk. The microencapsulation was performed with a matrix of Poly(lactide-co-glycolide), which is an FDA approved polymer, and thanks to the permeability of this and the wall of pathogenic bacteria, thymol at a concentration of 50 mg/ml, allowed inhibiting bacterial growth by disruption of the cytoplasmic membrane [89–91].

As demonstrated above, co-microencapsulation can also be used to preserve microorganisms and other types of oils that are non-aromatic but nutritional. This was demonstrated by Vaziri, A. S., Alemzadeh, I., & Vossoughi, M. in 2019. They evaluated the co-microencapsulation of probiotic *L. plantarum* and DHA, looking for a synergy between the two. Co-microencapsulation of probiotics may be useful to produce additives or supplements to help the population with gastrointestinal problems or chronic diseases related to the gastric pathway [92,93]. In addition, DHA oil has a great importance in the nutritional aspect being an essential fatty acid, so its consumption is necessary for the growth and functionality of brain cells, also endothelial cells, and leukocytes. However, there is an important factor that must be considered for its storage, and that is that this oil can oxidize very easily and quickly. Due to its oxidation, it can provide bad taste to the food in which it is contained, besides taking away all its benefits and even making it carcinogenic [94–96]. According to this work, these co-microencapsulates were added to orange juice with a pH of about 4. In a free environment, most of the *L. plantarum* cells could have survived, but the DHA could have been oxidized due to pH conditions and ambient light; in contrast, by co-microencapsulation, stability of the microorganism and the oil was achieved while retaining the benefits and nutrients [93].

## 6. Conclusion

In co-microencapsulation, in order to find the ideal matrix to protect the active compounds, as well as to find and/or develop a synergy between the active compounds, it is necessary for the researcher to explore and evaluate the encapsulating materials and active compounds available to him, taking into account the accessibility, characteristics and cost of each one of them, so that he can select those that suit his situation and are of interest to him.

For the selection of a co-microencapsulation method, the use of the lyophilization technique is recommended, due to the great versatility of the use of this technique in most encapsulation materials and active compounds, in turn, limits the degradation of the active compounds, and does not require an additional drying process as the coextrusion and coacervation techniques.

Lyophilization has the drawback of high equipment cost both at laboratory and industrial scale, so the spray-drying co-microencapsulation technique is also a good option, as scaling up production to industrial scale is more feasible. Like freeze-drying, it does not require an additional drying process, both co-microencapsulation techniques being fast. In the case of selecting the spray drying technique for co-microencapsulation, it is necessary to evaluate and select the ideal temperatures to carry out the process, since the high temperatures usually used in this technique can degrade the active compounds and the encapsulation materials, resulting in a low encapsulation efficiency of the active compounds, and a shorter shelf life of the microcapsules.

In addition, each of the methodologies used considers different operating conditions, so the use of experimental designs that allow the optimization of both the conditions and the composition of the encapsulating matrix will allow a better use of resources.

## 7. Future tendencies of the co-microencapsulation

Co-microencapsulation is a technique with a wide area of opportunity for future development, as it can be performed with almost any food grade material considered as GRAS, using it as encapsulating material or as active compound, such as dextrans, gum arabic, Persian gum, galactooligosaccharides, glucose, sucrose, lactose, maltodextrin, starch, vitamins, egg white and mannitol. The challenge of co-microencapsulation is to find the synergy between active compounds and encapsulating materials for further optimization and to make the generation of such microcapsules more efficient, thus reducing operating costs [6,11,25,39,97].

One of the most novel proposals would be the use of co-microencapsulation for more than two active compounds, where two probiotics are included together with a prebiotic. This would have the additive effect of two probiotics with different benefits each. It would also be possible to combine probiotics, a prebiotic and a polyphenol, to have the probiotic effect, the light fiber contribution of the prebiotic, as well as the antioxidant benefits of the polyphenols [Y. 6, 9, 21, 29]. It is even important to explore the prebiotic effect of polyphenols so that in an encapsulated system they can be incorporated to exert antioxidant, antimicrobial and anticancer effects together with the benefits that a probiotic can provide.

Microcapsules generated by co-microencapsulation, which have a greater beneficial activity for the organism can be used in a greater amount of food products to provide higher quality food, as well as it could be used as food supplements [6,21,25,75,79].

In any of their presentations these co-microencapsulates could provide fiber, probiotics, essential fatty acids, essential amino acids, antioxidants and pathogen antimicrobials in the same microcapsule; as described by Nami et al. in 2020, by adding microcapsules to a commercial orange juice without preservatives. The microcapsules contain *Lactococcus lactis* ABRIINW-N19 and inulin as active compounds, are enveloped by alginate and Persian gum, they reported that the juice added with the microcapsules had a higher amount of probiotics of those that were inoculated, in turn, Brix and malic acid were reduced, this without altering the pH, providing an alternative for probiotic intake with a lower amount of sugars [6,21,25,75,79].

The next step of co-microencapsulation is experimentation in in vivo models, to continue with human clinical trials, and to accept or deny the potential benefits of microcapsules in human life due to the potential to reduce the incidence and prevalence of chronic diseases such as diabetes mellitus, hypertension, obesity, metabolic syndrome, acute myocardial infarctions, cerebral vascular events, irritable bowel syndrome, gastritis and malnutrition; leading us to a new stage in the generation of new foods, food supplements and even therapeutic-grade foods in patients with gut microbiota dysbiosis [6, 9, 11, 14, 21, 25, 27, 29, 49, 79].

The generation of these novel foods, food supplements and therapeutic foods have the challenge of being produced on an industrial scale as most of them are not usually laboratory or pilot plant level foods, which are hardly marketed and distributed. This low production and distribution are multifactorial, with economic investment being the main limiting factor; researchers could seek to offer their products to food companies, looking for companies to include them among their products so that they can be marketed and distributed to a larger number of people.

## References

[1] Ban KM. Goal 2: Zero hunger, Goal 3: Good health and well-being, Goal 12: Responsible consumption and production. The Sustainable Development Goals Report, (pp. 14-17, 34-35). (New York, NY: United Nations Publications). 2016.

[2] Castañeda Guillot C. Probióticos, puesta al día: an update. Revista Cubana de Pediatría. 2018;90(2):286–298.

[3] U SDG. (2019). Sustainable development goals. The energy progress report. Tracking SDG, 7.

[4] Hassan A, Din AU, Zhu Y, et al. Anti-atherosclerotic effects of Lactobacillus plantarum ATCC 14917 in ApoE−/− mice through modulation of proinflammatory cytokines and oxidative stress. Appl Microbiol Biotechnol. 2020;104:6337–6350. 32472174 10.1007/s00253-020-10693-x

[5] Matiacevich S, Sáez C (2018). Encapsulación de aceite esencial de lemongrass en el desarrollo de ingredientes naturales en polvo para preservación de alimentos: una revisión.

[6] Raddatz GC, Menezes CRD. Microencapsulation and co-encapsulation of bioactive compounds for application in food: challenges and perspectives. Ciência Rural. 2021;51(3). DOI:10.1590/0103-8478cr20200616

[7] Ramirez Ramirez JC, Rosas Ulloa PETRA, and Velazquez Gonzalez MY, et al. Bacterias lácticas: importancia en alimentos y sus efectos en la salud. Revista Fuente CONACYT. 2011;2(7) .

[8] Tarifa MC, Piqueras CM, and Genovese DB, et al. Microencapsulación de Lactobacillus casei y Lactobacillus rhamnosus en partículas de microgel de pectina y pectina-inulina: efecto sobre la supervivencia bacteriana en condiciones de almacenamiento, 10th International Conference of Production Research Americas December 2020 Bahía Blanca, Argentina, 2021. (ICPR-Americas 2020), 1859–1863.

[9] Wang Y, Dong Z, Song D, et al. Efectos de los probióticos y prebióticos microencapsulados sobre el rendimiento del crecimiento, las capacidades antioxidantes, las funciones inmunes y la microflora cecal en pollos de engorde. Inmunología alimentaria y agrícola. 2018;29(1):859–869.

[10] Wu L, Wang W, Wu Z, et al. Effect of acid and alkali stress on extracellular metabolite profile of Lactobacillus plantarum ATCC 14917. J Basic Microbiol. 2020;60:722–729. 32452552 10.1002/jobm.202000203

[11] Zhu H, Mettu S, and Cavalieri F, et al. Ultrasonic microencapsulation of oil-soluble vitamins by hen egg white and green tea for fortification of food. Food Chemistry. 2021;353:129432. 33714120 10.1016/j.foodchem.2021.129432 PMC8164159

[12] Álvarez-García R. Efecto de los polifenoles sobre la microbiota intestinal en el síndrome metabólico. 2020.

[13] Arellano K, Vazquez J, Park H, et al. Safety evaluation and whole-genome annotation of Lactobacillus plantarum strains from different sources with special focus on isolates from green tea. Probiotics Antimicrob Proteins. 2020;12(3):1057–1070. 31786735 10.1007/s12602-019-09620-y

[14] Krasaekoopt WY, and Watcharapoka S. Effect of addition of inulin and galactooligosaccharide on the survival of microencapsulated probiotics in alginate beads coated with chitosan in simulated digestive system, yogurt and fruit juice. LWT-Ciencia y tecnología de los alimentos. 2014;57(2):761–766 doi:10.1016/j.lwt.2014.01.037.

[15] Zhang ZH, Peng H, Woo MW, et al. Preparation and characterization of whey protein isolate-chlorophyll microcapsules by spray drying: effect of WPI ratios on the physicochemical and antioxidant properties. J Food Eng. 2020;267:109729.

[16] Wu Y, Li S, Tao Y, et al. Fermentation of blueberry and blackberry juices using Lactobacillus plantarum, Streptococcus thermophilus and Bifidobacterium bifidum: growth of probiotics, metabolism of phenolics, antioxidant capacity in vitro and sensory evaluation. Food Chem. 2021;348:129083. 33517000 10.1016/j.foodchem.2021.129083

[17] Castromonte M, Wacyk J, Valenzuela C. Encapsulación de extractos antioxidantes desde sub-productos agroindustriales: una revisión. Revista chilena de nutrición. 2020;47(5):836–847.

[18] Castro-Torres AY. Evaluación del efecto prebiótico de polifenoles obtenidos mediante una tecnología híbrida a partir de cultivos, subproductos y recursos naturales poco valorados. Proyecto 2015-4-266936 SAGARPA-CONACyT. 2019.

[19] Colín-Cruz MA, Pimentel-González DJ, Carrillo-Navas H, et al. Co-encapsulation of bioactive compounds from blackberry juice and probiotic bacteria in biopolymeric matrices. LWT. 2019;110:94–101.

[20] Creus EG. Alimentos prebióticos y probióticos: la polémica científica sobre sus beneficios. Offarm: farmacia y sociedad. 2004;23(5):90–98.

[21] Favaro-Trindade CS, Patel B, Silva MP, et al. Microencapsulation as a tool to producing an extruded functional food. LWT. 2020;128:109433.

[22] Gasaly N, Riveros K, Gotteland M. Fitoquímicos: una nueva clase de prebióticos. Revista chilena de nutrición. 2020;47(2):317–327.

[23] Loyeau PA, Spotti MJ, Braber NV, et al. Microencapsulation of Bifidobacterium animalis subsp. lactis INL1 using whey proteins and dextrans conjugates as wall materials. Food Hydrocoll. 2018;85:129–135.

[24] Rodríguez-Barona S, Giraldo GI, Montes LM. Encapsulación de alimentos probióticos mediante liofilización en presencia de prebióticos. Información tecnológica. 2016;27(6):135–144.

[25] Ye Q, Georges N, Selomulya C. Microencapsulation of active ingredients in functional foods: from research stage to commercial food products. Trends Food SciTechnol. 2018;78:167–179.

[26] Ye Q, Georges N, Selomulya C. Microencapsulation of active ingredients in functional foods: from research stage to commercial food products. Trends Food SciTechnol. 2018;78:167–179.

[27] Eratte D, Dowling K, Barrow CJ, et al. In-vitro digestion of probiotic bacteria and omega-3 oil co-microencapsulated in whey protein isolate-gum Arabic complex coacervates. Food Chem. 2017;227:129–136. 28274412 10.1016/j.foodchem.2017.01.080

[28] Vaziri AS, Alemzadeh I, Vossoughi M, et al. Co-microencapsulation of Lactobacillus plantarum and DHA fatty acid in alginate-pectin-gelatin biocomposites. Carbohydr Polym. 2018;199:266–275. 30143129 10.1016/j.carbpol.2018.07.002

[29] Vázquez-Maldonado D, Espinosa-Solis V, Leyva-Porras C, et al. Preparation of spray-dried functional food: effect of adding Bacillus clausii bacteria as a co-microencapsulating agent on the conservation of resveratrol. Processes. 2020;8(7):849.

[30] Chen W, Wang H, Zhang K, et al. LiPhysicochemical properties and storage stability of microencapsulated DHA-rich oil with different wall materials. Appl Biochem Biotechnol. 2016;179(7):1129–1142. 27003283 10.1007/s12010-016-2054-3

[31] Wang W, He J, Pan D, et al. Metabolomics analysis of Lactobacillus plantarum ATCC 14917 adhesion activity under initial acid and alkali stress. PloS one. 2018;13(5):e0196231. 29795550 10.1371/journal.pone.0196231 PMC5967736

[32] López Córdoba AF (2012). *Desarrollo de sistemas de encapsulación compuestos para la protección de extractos antioxidantes de yerba mate* (Doctoral dissertation, Universidad Nacional de La Plata).

[33] Assadpour E, Jafari SM. Advances in spray-drying encapsulation of food bioactive ingredients: from microcapsules to nanocapsules. Annu Rev Food Sci Technol. 2019;10:103–131. 30649963 10.1146/annurev-food-032818-121641

[34] Prakash B, Kujur A, Yadav A, et al. Nanoencapsulation: an efficient technology to boost the antimicrobial potential of plant essential oils in food system. Food Control. 2018;89:1–11.

[35] Manuel Irache J. Micro- y nanoencapsualción de aditivos y otros compuestos de interés alimentario. In: 1er Congreso Nacional de Agroalimentación. Pamplona: Universidad de Navarra, España; 2011 May.

[36] Timilsena YP, Akanbi TO, Khalid N, et al. Complex coacervation: principles, mechanisms and applications in microencapsulation. Int J Biol Macromol. 2019;121:1276–1286. 30352231 10.1016/j.ijbiomac.2018.10.144

[37] Gharibzahedi SMT, Jafari SM. The importance of minerals in human nutrition: bioavailability, food fortification, processing effects and nanoencapsulation. Trends Food SciTechnol. 2017;62:119–132.

[38] Enache IM, Vasile AM, Enachi E, et al. Co-microencapsulation of anthocyanins from black currant extract and lactic acid bacteria in biopolymeric matrices. Molecules. 2020;25(7):1700. 10.3390/molecules25071700 PMC7181145 32276335

[39] Mahdavi SA, Jafari SM, Assadpoor E, et al. Microencapsulation optimization of natural anthocyanins with maltodextrin, gum Arabic and gelatin. Int J Biol Macromol. 2016;85:379–385. 26772915 10.1016/j.ijbiomac.2016.01.011

[40] Uyen NTT, Hamid ZAA, Tram NXT, et al. Fabricación de microesferas de alginato para la administración de fármacos: una revisión. Revista internacional de macromoléculas biológicas. 2020;153:1035–1046.

[41] Pisano R, Arsiccio A, Capozzi LC, et al. Achieving continuous manufacturing in lyophilization: technologies and approaches. Eur J Pharm Biopharm. 2019;142:265–279. 31252071 10.1016/j.ejpb.2019.06.027

[42] Ballesteros LF, Ramirez MJ, Orrego CE, et al. Encapsulation of antioxidant phenolic compounds extracted from spent coffee grounds by freeze-drying and spray-drying using different coating materials. Food Chem. 2017;237:623–631. 28764044 10.1016/j.foodchem.2017.05.142

[43] Poozesh S, Bilgili E. Scale-up of pharmaceutical spray drying using scale-up rules: a review. Int J Pharm. 2019;562:271–292. 30910632 10.1016/j.ijpharm.2019.03.047

[44] Agudelo J, Cano A, González-Martínez C, et al. Disaccharide incorporation to improve survival during storage of spray dried Lactobacillus rhamnosus in whey protein-maltodextrin carriers. J Funct Foods. 2017;37:416–423.

[45] Silva MP, Tulini FL, Martins E, et al. Comparison of extrusion and co-extrusion encapsulation techniques to protect *Lactobacillus acidophilus* LA3 in simulated gastrointestinal fluids. LWT. 2018;89:392–399.

[46] Yong AKL, Lai KW, Ghazali HM, et al. Microencapsulation of Bifidobacterium animalis subsp. lactis BB-12 with Mannitol. Asia-Pacific J Mol Biol Biotechnol. 2020;28(2):32–42.

[47] Eghbal N, and Choudhary R. Complex coacervation: encapsulation and controlled release of active agents in food systems. LWT. 2018;90:254–264.

[48] Sing CE. Development of the modern theory of polymeric complex coacervation. Adv Colloid Interface Sci. 2017;239:2–16. 27161661 10.1016/j.cis.2016.04.004

[49] de Almeida Paula D, Martins EMF, and de Almeida Costa N, et al. Use of gelatin and gum arabic for microencapsulation of probiotic cells from Lactobacillus plantarum by a dual process combining double emulsification followed by complex coacervation. International Journal of Biological Macromolecules. 2019;133:722–731 doi:10.1016/j.ijbiomac.2019.04.110 2019. 31002903

[50] Senturk Parreidt T, Müller K, and Schmid M. Alginate-Based Edible Films and Coatings for Food Packaging Applications. Foods. 2018;7(10):170 doi:10.3390/foods7100170. PMC6211027 30336642

[51] Shamah-Levy T, Vielma-Orozco E, Heredia-Hernández O, et al. Encuesta Nacional de Salud y Nutrición 2018-19: resultados Nacionales. Cuernavaca, México: Instituto Nacional de Salud Pública; 2020.

[52] Nami Y, Lornezhad G, and Kiani A, et al. Alginate-Persian Gum-Prebiotics microencapsulation impacts on the survival rate of Lactococcus lactis ABRIINW-N19 in orange juice. LWT. 2020;124:109190 doi:10.1016/j.lwt.2020.109190.

[53] Lin L, Regenstein JM, Lv S, et al. An overview of gelatin derived from aquatic animals: properties and modification. Trends Food SciTechnol. 2017;68:102–112.

[54] Le Bastard Q, Chapelet G, and Javaudin F, et al. The effects of inulin on gut microbial composition: a systematic review of evidence from human studies. European Journal of Clinical Microbiology & Infectious Diseases. 2020;39(3):403–413 doi:10.1007/s10096-019-03721-w. 31707507

[55] Shang HM, Zhou HZ, and Yang JY, et al. In vitro and in vivo antioxidant activities of inulin. PLOS ONE. 2018;13(2):e0192273 doi:10.1371/journal.pone.0192273. 29394273 PMC5796717

[56] Shoaib M, Shehzad A, Omar M, et al. Inulin: properties, health benefits and food applications. Carbohydr Polym. 2016;147:444–454. 27178951 10.1016/j.carbpol.2016.04.020

[57] Silva EK, Zabot GL, Bargas MA *et al*, et al. Microencapsulation of lipophilic bioactive compounds using prebiotic carbohydrates: Effect of the degree of inulin polymerization. Carbohydrates Polymers. 2016;152:775–783 doi:10.1016/j.carbpol.2016.07.066. 27516329

[58] Vandeputte D, Falony G, and Vieira-Silva S, et al. Prebiotic inulin-type fructans induce specific changes in the human gut microbiota. Gut. 2017;66(11):1968–1974 doi:10.1136/gutjnl-2016-313271. 28213610 PMC5739857

[59] Nunes G Lorenzoni, Etchepare M de, Cichoski A José, Zepka L Queiroz, Jacob Lopes E, Barin J Smanioto, Flores É Marlon, da Silva C de and de Menezes C Ragagnin. (2018). Inulin, hi-maize, and trehalose as thermal protectants for increasing viability of Lactobacillus acidophilus encapsulated by spray drying. LWT, 89 128–133. 10.1016/j.lwt.2017.10.032

[60] Lara-Espinoza C, Carvajal-Millán E, Balandrán-Quintana R, et al. Pectin and pectin-based composite materials: beyond food texture. Molecules. 2018;23(4):942. 10.3390/molecules23040942 PMC6017442 29670040

[61] Liang LI, Luo Y, Luo Y. Casein and pectin: structures, interactions, and applications. Trends Food SciTechnol. 2020;97:391–403.

[62] Naqash F, Masoodi FA, Rather SA, et al. Emerging concepts in the nutraceutical and functional properties of pectin—A Review. Carbohydr Polym. 2017;168:227–239. 28457445 10.1016/j.carbpol.2017.03.058

[63] Rehman A, Ahmad T, Aadil RM, et al. Pectin polymers as wall materials for the nano-encapsulation of bioactive compounds. Trends Food SciTechnol. 2019;90:35–46.

[64] Zhou Y, Zhu X, Zhang C, et al. Characterization of whey protein isolate and pectin composite film catalyzed by small laccase from *Streptomyces coelicolor*. Environ Technol Innovation. 2020;19:100999.

[65] Niu X, Liu A, Liu C, et al. Small Laccase from *Streptomyces coelicolor* catalyzed chitosan-pectin blending film for hazardous gas removal. Environ Technol Innovation. 2021;101690.

[66] Oancea A-M, Hasan M, Vasile AM, et al. Functional evaluation of microencapsulated anthocyanins from sour cherries skins extract in whey proteins isolate. LWT. 2018;95:129–134.

[67] Ordoñez-Gómez ES, Reátegui-Díaz D, Villanueva-Tiburcio JE. Polifenoles totales y capacidad antioxidante en cáscara y hojas de doce cítricos. Scientia Agropecuaria. 2018;9(1):113–121.

[68] Bakshi PS, Selvakumar D, Kadirvelu K, et al. Chitosan as an environment friendly biomaterial–a review on recent modifications and applications. Int J Biol Macromol. 2020;150:1072–1083. 31739057 10.1016/j.ijbiomac.2019.10.113

[69] Mujtaba M, Morsi RE, Kerch G, et al. Current advancements in chitosan-based film production for food technology; A review. Int J Biol Macromol. 2019;121:889–904. 30340012 10.1016/j.ijbiomac.2018.10.109

[70] Vallejo-Domínguez D, Rubio-Rosas E, Aguila-Almanza E, et al. Ultrasound in the deproteinization process for chitin and chitosan production. Ultrason Sonochem. 2021;72:105417. 33352467 10.1016/j.ultsonch.2020.105417 PMC7803815

[71] Qi PX, Xiao Y, Wickham ED. Changes in physical, chemical and functional properties of whey protein isolate (WPI) and sugar beet pectin (SBP) conjugates formed by controlled dry-heating. Food Hydrocoll. 2017;69:86–96.

[72] Quigley EM. Prebiotics and probiotics in digestive health. Clin Gastroenterol Hepatol. 2019;17(2):333–344. 30267869 10.1016/j.cgh.2018.09.028

[73] Quiñones M, Miguel M, Aleixandre A. Los polifenoles, compuestos de origen natural con efectos saludables sobre el sistema cardiovascular. Nutrición hospitalaria. 2012;27(1):76–89. 22566306 10.1590/S0212-16112012000100009

[74] Shang J, Liao M, Jin R, et al. Molecular Properties of flammulina velutipes polysaccharide–whey protein isolate (WPI) complexes via noncovalent interactions. Foods. 2021;10(1):1. 10.3390/foods10010001 PMC7821936 33374899

[75] Guillot CDC. Microbiota intestinal, probióticos y prebióticos. Enfermería investiga: investigación, vinculación, docencia y gestión. 2017;2(4):156–160.

[76] Huertas RAP. Bacterias ácido lácticas: papel funcional en los alimentos. Biotecnología en el sector agropecuario y agroindustrial. 2010;8(1):93–105.

[77] Fareez IM, Lim SM, Mishra RK, et al. Chitosan coated alginate-xanthan gum bead enhanced pH and thermotolerance of Lactobacillus plantarum LAB12. Int J Biol Macromol. 2015;72:1419–1428. 25450046 10.1016/j.ijbiomac.2014.10.054

[78] Instituto Nacional de Salud Pública. Encuesta Nacional de Salud y Nutrición 2018. Resultados de Coahuila. Cuernavaca, México: Instituto Nacional de Salud Pública; 2020.

[79] Valdovinos MA, Montijo E, Abreu AT, et al. Consenso mexicano sobre probióticos en gastroenterología. Revista de Gastroenterología de México. 2017;82(2):156–178. 28104319 10.1016/j.rgmx.2016.08.004

[80] Duar RM, Lin XB, Zheng J, et al. Lifestyles in transition: evolution and natural history of the genus Lactobacillus. FEMS Microbiol Rev. 2017;41(Supp_1):S27–S48. 28673043 10.1093/femsre/fux030

[81] Dueñas M, Fernández D, Hernández T, et al. Bioactive phenolic compounds of cowpeas (Vigna sinensis L). Modifications by fermentation with natural microflora and with Lactobacillus plantarum ATCC 14917. J Sci Food Agric. 2005;85(2):297–304.

[82] Mariño García A, Núñez Velázquez M, Barreto Penié J. Microbiota, probióticos, prebióticos y simbióticos. Acta Médica de Cuba. 2016;17(1).

[83] Márquez NO. Efectos Del uso de prebióticos y probióticos en la enfermedad de alzheimer effects of the use of prebiotics and probiotics in Alzheimer’s Disease. Actualización en Nutrición. 2020;21(2):65–70.

[84] Murray PR, Rosenthal , KS, Pfaller, MA. Human Microbiome in Health and Disease . Medical Microbiology. Philadlphia, PA: ELSEVIER; 2016;5–10. ISBN: 978-0-323-29956-5.

[85] Valencia Avilés E, Figueroa I, Sosa Martínez E, Bartolomé-Camacho, MC, et al. Polifenoles: propiedades antioxidantes y toxicológicas. Revista de la Facultad de Ciencias Químicas. 2017;16:15–29. ISBN: 1390-1869 .

[86] Peñarrieta JM, Tejeda L, and Mollinedo P, et al. Phenolic compounds in food. Revista Boliviana de Química. 2014;31(2):68–81.

[87] Enache IM, Vasile AM, Enachi E, et al. Co-microencapsulation of anthocyanins from cornelian cherry fruits and lactic acid bacteria in biopolymeric matrices by freeze-drying: evidences on functional properties and applications in food. Polymers. 2020;12(4):906. 10.3390/polym12040906 PMC7240423 32295223

[88] Enache IM, Vasile AM, Enachi E, et al. Co-microencapsulation of anthocyanins from black currant extract and lactic acid bacteria in biopolymeric matrices by freeze-drying. Molecules. 2020;25:1700. 10.3390/molecules25071700 PMC7181145 32276335

[89] Jain RA. The manufacturing techniques of various drug loaded biodegradable poly (lactide-co-glycolide)(PLGA) devices. Biomaterials. 2000;21(23):2475–2490. 11055295 10.1016/s0142-9612(00)00115-0

[90] Marchese A, Orhan IE, Daglia M, et al. Antibacterial and antifungal activities of thymol: a brief review of the literature. Food Chem. 2016;210:402–414. 27211664 10.1016/j.foodchem.2016.04.111

[91] Zhu Z, Min T, Zhang X, et al. Microencapsulation of thymol in poly (lactide-co-glycolide)(PLGA): physical and antibacterial properties. Materials. 2019;12(7):1133. 10.3390/ma12071133 PMC6480635 30959946

[92] Pandey P, Mishra HN. Co-microencapsulation of γ-aminobutyric acid (GABA) and probiotic bacteria in thermostable and biocompatible exopolysaccharides matrix. LWT. 2021;136:110293.

[93] Vaziri AS, Alemzadeh I, Vossoughi M. Survivability and oxidative stability of co-microencapsulated L. Plantarum PTCC 1058 and DHA as a juice carrier. Food Biosci. 2019;32:100460.

[94] Ghasemi Fard S, Wang F, Sinclair AJ, et al. How does high DHA fish oil affect health? A systematic review of evidence. Crit Rev Food Sci Nutr. 2019;59(11):1684–1727. 29494205 10.1080/10408398.2018.1425978

[95] Gonzabay Cuadrado CS, Lindao Bararata CS (2019). *Determinación de polifenoles totales y actividad antioxidante del extracto metanólico de la cáscara de sandía (*Citrullus lanatus*) variedad Charleston Grey* (Doctoral dissertation, Universidad de Guayaquil. Facultad de Ciencias Químicas).

[96] Shen Y, Lu T, Liu XY, et al. Improving the oxidative stability and lengthening the shelf life of DHA algae oil with composite antioxidants. Food Chem. 2020;313:126139. 31927203 10.1016/j.foodchem.2019.126139

[97] Tantratian S, Pradeamchai M. Select a protective agent for encapsulation of Lactobacillus plantarum. LWT. 2020;123:109075.

